# Mobile Mental Health in Women’s Community-Based Organizations: Protocol for a Pilot Randomized Controlled Trial

**DOI:** 10.2196/42919

**Published:** 2023-02-08

**Authors:** Amritha Bhat, B Ramakrishna Goud, Bharat Kalidindi, Johnson Pradeep Ruben, Dhinagaran Devadass, Abijeet Waghmare, Pamela Y Collins, Tony Raj, Krishnamachari Srinivasan

**Affiliations:** 1 Department of Psychiatry and Behavioral Sciences University of Washington Seattle, WA United States; 2 St John’s Medical College Bengaluru India; 3 St John's Research Institute Bengaluru India; 4 Department of Global Health University of Washington Seattle, WA United States

**Keywords:** mobile mental health, women, community-based, depression, rural, stepped care

## Abstract

**Background:**

Of every 10 women in rural India, 1 suffers from a common mental disorder such as depression, and untreated depression is associated with significant morbidity and mortality. Several factors lead to a large treatment gap, specifically for women in rural India, including stigma, lack of provider mental health workforce, and travel times. There is an urgent need to improve the rates of detection and treatment of depression among women in rural India without overburdening the scarce mental health resources.

**Objective:**

We propose to develop, test, and deploy a mental health app, MITHRA (Multiuser Interactive Health Response Application), for depression screening and brief intervention, designed for use in women’s self-help groups (SHGs) in rural India.

**Methods:**

We will use focus groups with SHG members and community health workers to guide the initial development of the app, followed by iterative modification based on input from a participatory design group consisting of proposed end users of the app (SHG members). The final version of the app will then be deployed for testing in a pilot cluster randomized trial, with 3 SHGs randomized to receive the app and 3 to receive enhanced care as usual.

**Results:**

This study was funded in June 2021. As of September 2022, we have completed both focus groups, 1 participatory design group, and app development.

**Conclusions:**

Delivering app-based depression screening and treatment in community settings such as SHGs can address stigma and transportation-related barriers to access to depression care and overcome cultural and contextual barriers to mobile health use. It can also address the mental health workforce shortage. If we find that the MITHRA approach is feasible, we will test the implementation and effectiveness of MITHRA in multiple SHGs across India in a larger randomized controlled trial. This approach of leveraging community-based organizations to improve the reach of depression screening and treatment is applicable in rural and underserved areas across the globe.

**International Registered Report Identifier (IRRID):**

DERR1-10.2196/42919

## Introduction

### Background

Treatment rates for major depression range from 23% in high-income countries to 3% in low- and lower-middle–income countries [1]. The treatment gap is higher in rural populations, especially among women [2], despite the fact that 1 in 10 women in rural India has a common mental disorder (CMD) such as depression [3]. Untreated depression is associated with significant morbidity and mortality [4,5]. Risk factors that are more prevalent in rural areas, such as low levels of education, financial difficulties, intimate partner violence, and alcohol use in the husband [6], directly influence the outcomes of CMD and help seeking, creating barriers to care [7]. Factors contributing to the treatment gap include the dearth of psychiatrists and other mental health professionals [8,9], poor transportation infrastructure, and stigma, leading to patients not accessing care [10]. Integrating a mental health program into rural primary health centers can improve access but cannot fully address concerns about high rates of medication nonadherence and treatment dropouts [11]. In previous studies [12] and in our recent qualitative analysis [13], women cite travel times and inability to take time off from work as a significant barrier to seeking mental health care in the primary health center (PHC), and as a reason for treatment discontinuation. This barrier is relevant throughout the country, as although India’s District Mental Health Program aims to provide community-based accessible mental health care, 40% of patients still travel more than 10 km to access mental health services [14].

Partnering with community-based organizations (CBOs) is one way to address barriers to accessing mental health treatments [15]. CBOs for women (called “mahila mandals” or “sthree shakthi”–women power or self-help groups [SHG]) are instrumental in increasing women’s empowerment and participation in microfinancing systems in rural India. Each group consists of about 15 to 20 women members from a defined geographic area who meet regularly (weekly, biweekly, or monthly) [16]. SHG activities include pooling of resources and extending microcredit, discussion of social issues, and supporting skill development and education [17]. Rates of positive depression screens among SHG attendees range from 10% to 13% [18].

Even mild depression is associated with decreased work productivity and presenteeism (working while sick) [19], and costs associated with presenteeism tend to be 5-10 times higher than those associated with absenteeism across different countries [20]. Identifying and treating mild depressive symptoms adequately can prevent major depression [21]. There is therefore an urgent need and opportunity to identify and treat mild-to-moderate depression without increasing burden on the strained health care system. Self-administered treatments are effective in reducing mild-to-moderate depressive symptoms [22]. Self-administered treatments include bibliotherapy and online or app-based online psychotherapy modules, and when combined with regular monitoring of symptoms, can be an effective treatment for mild-to-moderate depression. There is a strong evidence base for the stepped care approach to mental health treatment in which treatment begins with low-intensity interventions, which are then stepped up if symptoms persist [23] or if the patient declines the first-line treatment. Patients with high-level symptomatology are triaged to more intense interventions at the outset. This approach is especially useful in leveraging the available workforce in resource-constrained settings [24,25]. App-based depression interventions can improve access to treatment for CMD due to ease of access and availability even in resource-poor settings [26,27]. India has the second largest wireless communication subscriber base in the world [28], and most people have access to a mobile phone and network coverage [29]. However, there are differences in usage patterns that prompted us to develop a multiuser app on a tablet rather than an app designed to be used on a personal cell phone. For example, in rural India, while 87% of people own a cell phone, only 14% of them use text messaging [26], perhaps because of the high rate of illiteracy [30]. In addition, although women in rural India do report owning a mobile phone, this is often a shared family phone that is with the husband for most of the day [13].

We propose, in this funded grant, to develop, with end user feedback, a multiuser mobile app (MITHRA [Multiuser Interactive Health Response Application]=“friend” in Kannada, the regional language) for use in CBOs in rural India, to support the identification, initial treatment, and referral of women with depression. Our app will account for barriers such as illiteracy and lack of access to a personal mobile device. Our participatory approach will ensure that the app is responsive to local perceived needs by obtaining end user or consumer feedback at every stage of development [13]. The app will deliver education about depression and activity scheduling, based on the Healthy Activity Program (HAP), which is an evidence-based intervention delivered from 6 to 9 sessions; is acceptable, efficacious, and cost-effective in the treatment of depression; and has been tested in rural India [31]. Moreover, it is effective in the treatment of moderate-to-severe depression [32] and can benefit mildly depressed individuals who may not require the involvement of a mental health professional. Mild-to-moderate symptoms will be addressed by delivering the HAP via multimedia modules. This ensures that most women with mild-to-moderate depression receive initial treatment without having to overcome transportation barriers. Those with severe or nonresponsive symptoms will be directed to the PHC for assessment and treatment.

### Conceptual Model

**Figure 1:**
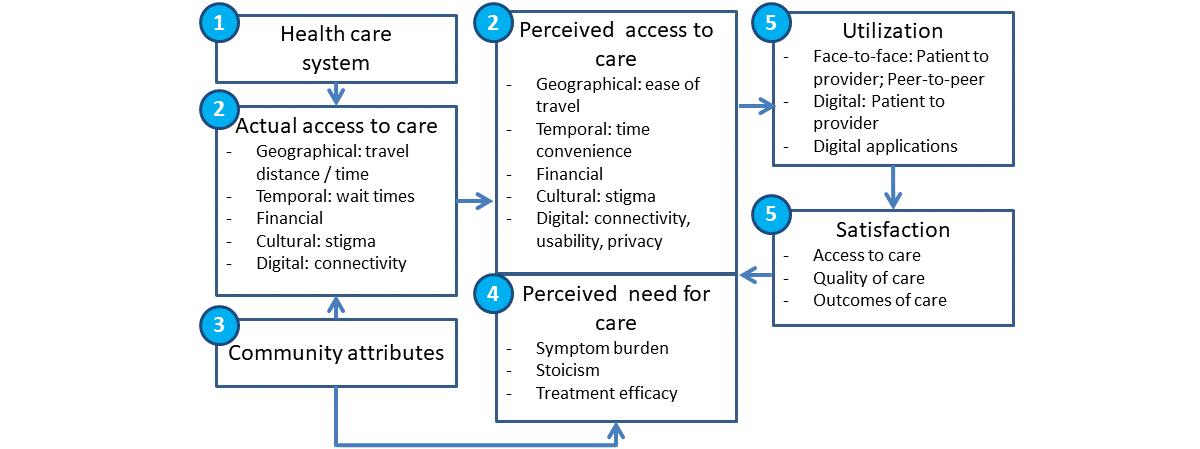
MITHRA conceptual model. 1. MITHRA will refer to Primary Health Center women with severe symptoms or nonresponse. 2. MITHRA provides depression screening and tracking in the community. 3. MITHRA leverages scheduled gatherings of women from the same community. 4. Education delivered through MITHRA will influence perceived need for care 5. MITHRA will potentially improve utilization rates and satisfaction with care.

Our conceptual model is based on the access to care model by Fortney et al [33] (Figure 1). MITHRA targets each of the barriers to access to care (actual and perceived) commonly identified by patients and integrates them into both the health care system and the community. By using non–encounter-based screening, tracking, and low-intensity interventions, MITHRA improves access among women for whom stigma and travel times are a barrier. By providing education about depression, it addresses the perceived need for care among women who may not be proactive in seeking mental health care and supports adherence among those who initiate care. By integrating into existing social systems such as SHGs and using existing health care systems such as community health workers (CHWs) and the PHC, MITHRA will efficiently increase the scalability and reach of mental health treatments.

## Methods

### Overview

This study will be conducted in two phases—user-centered app development in Phase 1 and a pilot cluster randomized controlled trial (RCT) in Phase 2. The study site will be within the Anekal taluk, including 10 villages with functional SHGs with a total population of 9724 [34]. Focus groups and individual interviews, usability testing, and deployment of the tablet-based apps for the pilot RCT will all be conducted in these SHGs.

### Study Population

In Phase 1, during app development, we will recruit women SHG participants aged 18 to 59 years and residents of the village (ie, not a guest attendee at the SHG) for focus groups. We will also recruit community health workers and administrators of the SHGs who have their current role for at least 6 months. Those who are unable to participate in the informed consent discussion will be excluded. In Phase 2, for the pilot RCT, we will recruit women aged 18 to 59 years, who are residents of the village (ie, not a guest attendee) and are planning to attend SHG meetings regularly. Women who have been diagnosed with a severe mental illness such as bipolar disorder or schizophrenia, those who have had a suicide attempt or severe alcohol or substance use in the past 6 months, and those who are unable to participate in the informed consent discussion will be excluded from the pilot RCT.

### Focus Groups for App Development

**Figure 2:**
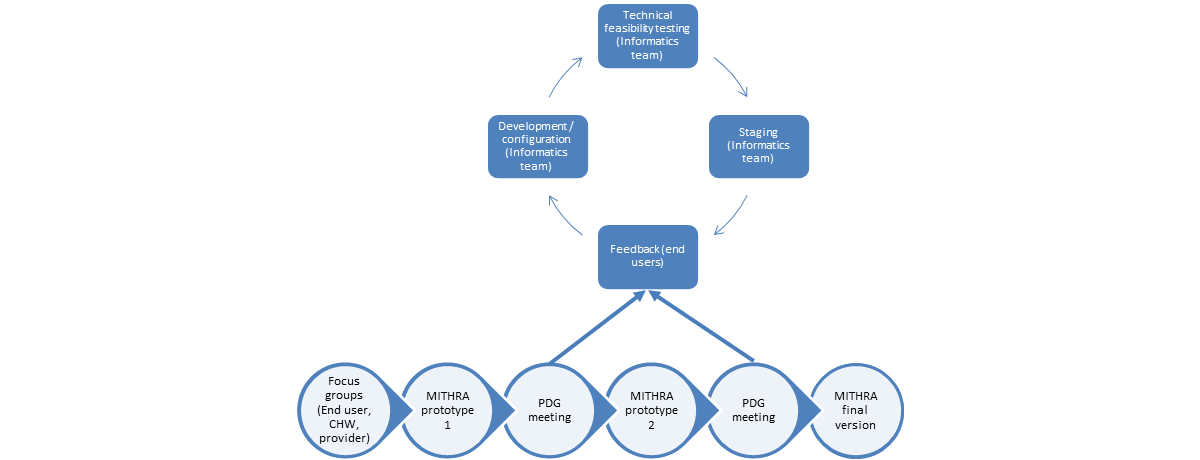
Stages of development of the MITHRA (Multiuser Interactive Health Response Application) app. CHW = Community Health Worker; PDG = Participatory Design Group (CHWs and women who attend CBO meetings).

App development will follow a user-centered iterative design approach (Figure 2). The process of administering the assessment questions and HAP modules will be based on the inputs provided by the SHG participants through the focus groups. One focus group will include community health workers (CHWs) and SHG administrators, and 1 will include SHG participants. We will develop interview guides [35,36] based on the acceptability on the interventions framework [37]. We will include questions on specifications such as simplified touchscreen, length of modules to be viewed, and women’s preferences regarding viewing HAP modules at home or at the SHG. We will record and transcribe focus group content and analyze it using Dedoose (SocioCultural Research Consultants), a qualitative analysis software. Using a thematic content analysis approach, we will identify common themes to help guide app development.

Next, we will enroll 5 to 6 members (including CHWs and SHG participants, representing the end users of the proposed app) from the focus groups into a participatory design group (PDG). During Phase 1, the team will build a prototype for the app, which includes video content based on the HAP modules, iteratively modifying the content and interface based on discussions with the PDG. We will complete the standard life cycle of software development, which includes development and configuration. We will then deploy the working prototype on the staging environment and complete further configurative iterations based on technical feasibility testing conducted with the PDG. Once iterative modifications are complete, the final version of the app will be deployed on the production environment for the RCT.

#### Data Analysis

We will record, transcribe, and translate the focus group content and analyze it using Dedoose. Using thematic content analysis, we will identify common themes to help guide app development. For the PDGs, we will collect end-user feedback on app prototypes until saturation of issues raised in PDG meetings is reached.

#### Description of MITHRA

The MITHRA app will be available on tablets placed in SHGs in an assigned private place. Each SHG will be provided with 2-3 tablets. Women can log in to MITHRA with a fingerprint-secure, single-user sign-on to complete the depression screening using the Patient Health Questionnaire-9 (PHQ-9). On completion of the PHQ-9, each woman will receive a prompt based on her total score. Those who score <5 will receive a prompt to watch general information on depression; those who score 5 or higher will watch select HAP modules, which will be short interactive multimedia-based modules, each 10 to 15 minutes long (or of other duration, based on findings from the focus group). They will also receive information on how to seek mental health care. Users can unlock virtual reward points or badges upon completion of the required questionnaires, modules, and activities. Any woman who scores anything other than 0 on question 9 of the PHQ-9 (suicidal ideation item) will be asked to call the CHW associated with that SHG. For these women, the app will also trigger an alert to the CHW, who will immediately contact the SHG administrator on site and call the patient to complete a risk assessment based on the suicidal ideation protocol [38].

The app will include a dashboard for the study team to access participant summary data and provide necessary alerts. Offline capabilities will ensure seamless use in areas with low to no internet connectivity. Data synchronization will occur at times of network connectivity, and the study team will ensure periodic synchronization of data between the study devices and servers hosted at the study team headquarters in India to ensure data security and to align with the institutional review board guidelines.

### Pilot RCT

Once app development is complete, we will randomize 3 SHGs to use MITHRA and 3 SHGs to enhanced usual care (EUC). Randomization will be clustered to account for distance from the PHC. In SHGs randomized to the intervention (MITHRA), a CHW will encourage women to use the MITHRA app. At each app use, women will complete the PHQ-9 screening and modules based on their individual depression score. Women typically attend SHG meetings 2-3 times a month, and the use of MITHRA at every attendance will be encouraged.

In the EUC SHGs, CHWs will offer monthly 45-minute group education regarding the symptoms of depression. The CHWs will in turn be trained in depression, screening and common treatments, resources, and referrals by study investigators to help support group education. In both MITHRA and EUC SHGs, CHWs will conduct community outreach to encourage women to attend meetings, as depression can lead to amotivation, and women with depression may be less likely to attend SHG meetings. CHWs will maintain rosters of SHG attendees at MITHRA SHGs and EUC SHGs. In monthly review sessions with the study investigators, the CHWs will review questionnaire completion and scores and directly contact the woman (by phone or home visit or at a SHG meeting) if there is a need to step up care beyond that advised by the app. We will aim to recruit approximately 60 women across 3 SHGs. The total number of participants will depend on the number of members in each enrolled SHG, which varies. We estimate an average of 10 in each SHG, or approximately 30 each in the intervention and control arms.

At recruitment, 3 months, and 6 months, a research assistant blinded to randomization status will administer outcome assessments in person or over the telephone. To avoid unblinding of SHG randomization status (eg, the presence of tablets in the SHGs), research assessments will be carried out by phone or in the PHC.

### Outcome Measures

The outcomes measures can be listed as follows: (1) app usage rates—we will obtain data on women's rates of use of the MITHRA app; (2) depression measured by Quick Inventory of Depressive Symptoms [39] at baseline, 3 months, and 6 months. Quick Inventory of Depressive Symptoms is a measure of depression symptoms severity that is sensitive to change; (3) behavioral activation—Behavioural Activation Depression Scale [40]. We will administer the Behavioural Activation Depression Scale to all women to calculate adherence to behavioral activation recommendations (for women enrolled in the intervention arm) and to measure degree of behavioral activation (for women enrolled in the EUC arm); (4) mental health services use—we will obtain information on rates of utilization of mental health services, including number of contacts with mental health providers and details of medications taken; and (5) System Usability Scale (SUS) [41], which is a widely used validated 10-item Likert scale that can be used to determine the usability of a wide variety of products and services, including hardware, software, mobile devices, websites, and applications, and it will be measured at the end of app usage from each woman enrolled in the intervention arm. Scores are “normalized” to produce a percentile ranking. Based on research, a SUS score of above 68 would be considered above average, and anything below 68 is below average.

### Data Analysis

We will descriptively summarize rates of initiation and continued use of the app in MITHRA sites. Usage rate calculations will be enabled by the single-user sign-on. We will also collect data on the usability of the app by administering the SUS to users of the app at 3 months and 6 months. For our primary outcome measures, we will perform intent-to-treat analyses. Although we expect that randomization will be successful and the 2 groups will be similar, we will compare baseline variables for differences, and in our analyses, control for variables were found to be significantly different between the 2 groups. We will use these preliminary data to examine the feasibility of randomization and measurement, as well as a methodological approach for a future larger trial, and initial signals regarding efficacy of the intervention.

### Ethical Considerations

This study has been reviewed and approved by the University of Washington Institutional Review Board and the Institutional Ethics Committee at St. John’s Medical College (approval number STUDY 00010415). All participants will be given adequate opportunity to ask for clarifications on study participation and procedures and will be recruited only after they provide informed consent; for those who cannot read, we will explain the study details and obtain consent by thumbprint and by recording verbal consent. A unique identifying code for each participant’s data will maintain confidentiality. Any forms with identifying information, such as the informed consent forms and participant list with assigned identification numbers, will be kept separate from study data in locked cabinets or on password-protected computers. Encryption will be used wherever data are transferred. At the end of the data retention period, all patient identifiers will be deleted in compliance with Health Insurance Portability and Accountability Act regulations. The app will be available on a tablet in the CBO, in a private location with headsets to maintain privacy. Each participant will be given a unique secure single-user sign on (which can be signed in using fingerprint for illiterate participants), thereby ensuring privacy of their information on this multiple-user app. All participants will receive Rs 200 (approximately $3) for participation. This amount represents a payment for the time and potential stress associated with the study procedures, which is comparable with other similar studies at this location and commensurate with the average income in the region, ensuring that consent is not unduly influenced by financial consideration.

## Results

This study was funded in June 2021. As of September 2022, we have completed both focus groups, 1 PDG, and the development of app wire frames.

## Discussion

### Principal Findings

The use of app-based screening in an SHG on a tablet that can be used by multiple women is in line with the description of mobile instruments as a “collectivist machine” rather than an individuality-enhancing device in rural areas of India [42]. Leveraging SHG meetings helps address the stigma and transportation-related barriers to access to depression care and overcomes the cultural and contextual barriers to mobile health use. MITHRA will support population-level symptom surveillance and tracking, monitoring adherence to recommendations, health education, and communication between patient and health worker. In addition, it can help promote awareness in the community about mental illness and help reduce stigma [43]. App-based delivery of brief psychotherapeutic interventions can address the mental health workforce shortage. In this study, we propose to iteratively develop an app to screen for depression and deliver brief psychological interventions in women’s CBO. We have successfully completed focus groups with women and administrators to inform the development of the app.

### Limitations

It is possible that we will encounter difficulties in developing an app that is audio enabled. However, the informatics team has substantial experience developing similar health care apps and will be able to troubleshoot accordingly. Participating women may not be enthusiastic about the use of the app. As we include input from potential users from the initial stages of app development, we hope to incorporate in our design specific features that women identify as desirable. EUC CBOs will only receive depression education from CHWs; however, this is still an enhancement compared to usual care, which is that these CBOs do not typically have depression-screening or health-education sessions.

### Impact and Future Directions

If we find that the MITHRA approach is feasible, we plan to further test the implementation and effectiveness of MITHRA in multiple CBOs across India. In India, women’s mental health is especially relevant to the health of the entire family as they play a multidimensional role in the family. The benefits of adequately treating depression in women will therefore accrue in their family as well. In the future, similar apps can be deployed in men’s CBOs. This approach of leveraging CBOs to improve the reach of depression screening and treatment is also applicable in rural and underserved areas across the globe. For example, in the United States, many low-income women receive support from CBOs such as Women’s Infant and Children [44] and other nutritional programs and peer-support groups, and these might be appropriate platforms for app-based screening and stepped care treatment.

The next steps could include examining the feasibility of making telepsychiatry available for women with severe depressive symptoms or those who do not respond to the first-line app-based treatment. Since the onset of the COVID-19 pandemic, telemedicine is being used in many settings and is acceptable to patients. There is emerging evidence for the feasibility of telepsychiatric consultations to district hospitals from academic centers [45], and this approach could be extended to examine the feasibility and effectiveness of telepsychiatry to SHGs. If the delivery of app-based screening and brief psychological interventions is found to be feasible in this study, we will test the effectiveness of this approach in a large RCT.

## Abbreviations

CBO: community-based organization
CHW: community health worker
CMD: common mental disorder
EUC: enhanced usual care
HAP: Healthy Activity Program
MITHRA: Multiuser Interactive Health Response Application
PDG: participatory design group
PHC: primary health center
PHQ: Patient Health Questionnaire
RCT: randomized controlled trial
SHG: self-help group
SUS: System Usability Scale

## Data Availability

The data sets generated during and/or analyzed during the current study will be available from the corresponding author on reasonable request.
